# Evolution of gene regulation of pluripotency - the case for wiki tracks at genome browsers

**DOI:** 10.1186/1745-6150-5-67

**Published:** 2010-12-29

**Authors:** Georg Fuellen, Stephan Struckmann

**Affiliations:** 1 Institute for Biostatistics and Informatics in Medicine and Ageing Research - IBIMA, University of Rostock, Medical Faculty, Ernst-Heydemann-Str. 8, 18057 Rostock, Germany; 2 Institute for Mathematics and Computer Science, University of Greifswald, Walther-Rathenau-Straße 47, 17487 Greifswald

## Abstract

**Background:**

Experimentally validated data on gene regulation are hard to obtain. In particular, information about transcription factor binding sites in regulatory regions are scattered around in the literature. This impedes their systematic in-context analysis, e.g. the inference of their conservation in evolutionary history.

**Results:**

We demonstrate the power of integrative bioinformatics by including curated transcription factor binding site information into the UCSC genome browser, using wiki and custom tracks, which enable easy publication of annotation data. Data integration allows to investigate the evolution of gene regulation of the pluripotency-associated genes Oct4, Sox2 and Nanog. For the first time, experimentally validated transcription factor binding sites in the regulatory regions of all three genes were assembled together based on manual curation of data from 39 publications. Using the UCSC genome browser, these data were then visualized in the context of multi-species conservation based on genomic alignment. We confirm previous hypotheses regarding the evolutionary age of specific regulatory patterns, establishing their "deep homology". We also confirm some other principles of Carroll's "Genetic theory of Morphological Evolution", such as "mosaic pleiotropy", exemplified by the dual role of Sox2 reflected in its regulatory region.

**Conclusions:**

We were able to elucidate some aspects of the evolution of gene regulation for three genes associated with pluripotency. Based on the expected return on investment for the community, we encourage other scientists to contribute experimental data on gene regulation (original work as well as data collected for reviews) to the UCSC system, to enable studies of the evolution of gene regulation on a large scale, and to report their findings.

**Reviewers:**

This article was reviewed by Dr. Gustavo Glusman and Dr. Juan Caballero, Institute for Systems Biology, Seattle, USA (nominated by Dr. Doron Lancet, Department of Molecular Genetics, Weizmann Institute of Science, Rehovot, Israel), Dr. Niels Grabe, TIGA Center (BIOQUANT) and Medical Systems Biology Group, Institute of Medical Biometry and Informatics, University Hospital Heidelberg, Germany (nominated by Dr. Mikhail Gelfand, Department of Bioinformatics, Institute of Information Transfer Problems, Russian Academy of Science, Moscow, Russian Federation) and Dr. Franz-Josef Müller, Center for Regenerative Medicine, The Scripps Research Institute, La Jolla, CA, USA and University Hospital for Psychiatry and Psychotherapy (part of ZIP gGmbH), University of Kiel, Germany (nominated by Dr. Trey Ideker, University of California, San Diego, La Jolla CA, United States).

## Background

Inferring the evolution of gene regulation is a complex bioinformatics task. Over the last years, it became evident that the degree of conservation of gene regulatory elements had been overestimated in the past [1-3]. However, this renders the exceptions all the more interesting: the lower the extent of conservation of regulatory elements, the more important the few elements that are conserved. Therefore, this paper discusses some biological background, theoretical principles and bioinformatics approaches to investigate the evolution of gene regulation, using three regulators of the cellular state of pluripotency as an example and revealing new insights into evolution of pluripotency.

In summary, we wish to:

1) Exemplify how the UCSC browser can be used to investigate the evolution of gene regulation.

2) Exemplify how the Wiki track at UCSC could be used to support such investigations by a large-scale community effort.

3) Report the results we obtained from our study of the evolution of gene regulation of three specific genes.

4) Put our results into a wider, general context by referring to Carroll's theoretical work.

### Gene Regulation and its Evolution

Strands of DNA include transcribed parts (genes), which are often used as blueprints for proteins, and 'regulatory elements', which decide in part about the timing and the amount of transcription [4]. If transcription factors bind to (some of) these elements, the amount of transcription may be altered. The elements can be organized into so-called modules, often termed cis-regulatory modules. These are usually bound by transcription factor complexes called 'enhanceosomes'. The typical regulatory region of a gene includes an array of cis-regulatory modules, usually consisting of sets of transcription factor binding sites (TFBS). Next to the transcription start site are the core and the proximal promoter (up to 250 base pairs), followed by the distal elements (the latter are more than 250 base pairs away from the transcription start site) [4]. The network of transcription factors and other regulators, together with the cis-regulatory modules of TFBSs and other regulatory elements on the DNA level, form the "gene regulatory network". Evolution of gene regulation is concerned with the evolution of the gene regulatory network [5-9]. Many regulatory elements evolve due to mutations, insertions and deletions of nucleotides, by selection, duplication, inversion, translocation or by random drift, or due to transposable elements. [1]. Their volatility can lead to high binding site turnover. Nevertheless, the evolution of some regulatory elements can be traced back to the origin of the vertebrate lineage [10].

### Principles of the Evolution of Gene Regulatory Networks

Evolution of gene regulatory networks for developmental processes should follow some general principles, from a theoretical point of view, as formulated by Carroll in a recent paper [11]. Regulators play a role in a number of different processes, following the principles of "*Mosaic pleiotropy" (the same proteins contribute to different developmental processes and body structures)*, and "*Heterotopy" (changes in spatial regulation are associated with morphological divergence)*. Both, transcription factor binding and gene expression reflect these principles, which tend to complicate computational inferences. Such inferences are possible, however, and they rely on four other principles observed by Carroll. *"Ancestral genetic complexity" *is a necessary condition: without it, there would not be a rich structure in ancestral gene regulatory networks and complexity would have evolved independently in recent lineages. The principles of *"Deep homology", "Functional equivalence of distant homologs"*, and *"Infrequent toolkit gene duplication" *are necessary as well, because even if ancestral complexity exists, it is only detectable, if it is conserved in recent lineages. In line with the complexity of the processes to be organized by the transcription factors and their target genes, the network of transcription factors and target genes must be large (*"Vast regulatory networks"*, Carroll). Finally, Carroll's principle of *"Modularity of cis-regulatory elements" *is a consequence of the pleiotropy of transcription factors, which affect their targets by grouping and binding together in a combinatorial context-dependent fashion. Genes that have to be regulated synchronously are expected to share some or all of their cis-regulatory elements [11]. Investigating the whole complex network of gene regulation in its entirety is a challenge. Concentrating on the subnetwork of early development, the challenge becomes smaller, even though due to the pleiotropy, subnetworks in general are neither independent, nor disjoint. [11].

### Evolutionary Bioinformatics of Gene Regulation

Bioinformatics tools and software for estimating, analyzing and/or visualizing the evolution of gene regulation are rare, because data are scarce [12] . In particular, sequence motifs describing TFBSs as parts of regulatory elements have low overall information content (binding sites feature a length of 4-20 bases, approximately), making their reliable in-silico detection difficult. Many transcription factor binding site prediction tools exploit libraries of known binding motifs *and *evolutionary conservation, and usually they infer sets of related sites (cis-regulatory modules). Assuming that conservation goes with functional importance, "phylogenetic profiling" and related methods [13-22] suggest that predicted binding sites are the more likely to be functional, the more conserved they are. The integrated analysis of the evolution of cis-regulatory modules and the network of regulators is in its infancy. By assembling experimentally validated TFBS information for a specific set of genes, we wish to contribute data that is useful for the development of methods and software towards this aim, and we hope that other researchers will follow suit, in a community/wiki effort.

### Gene Regulation in Stem Cells

Stem cell research is currently one of the most active areas in molecular biology and biomedicine, based in part on recent breakthroughs in generating 'induced pluripotent stem cells' (iPS cells) from somatic cells like fibroblasts (reviewed in [23,24]). Such a 'reprogramming' of differentiated cells into 'pluripotent' ones is possible by directly manipulating the pluripotency-related gene regulatory network [25] of the cell, confronting the differentiated cell with artificial amounts of key transcription factors such as Oct4 (also known as Pou5f1), Sox2 and Nanog. These 'ectopic' factors then re-direct the overall network of interaction and regulation. Redirection yields a state very close to the 'embryonic state'. In fact, mice can be obtained in which part (or even all) of their cells derive from the manipulated somatic cells [26]. Understanding the evolution of the gene regulatory network underlying stemness, or 'pluripotency', may give valuable guidance in improving reprogramming technology, highlighting similarities and differences across species, for example between model organisms and human.

However, data on pluripotency-related gene regulation are scattered around in the literature, and it takes a lot of manual effort to extract validated regulatory information from it. Because most papers lack genomic coordinates, it is not straightforward to obtain transcription factor binding sites with precise genomic location. Only with such precision, studies of their evolution become possible and these sites can be studied in the context of the wealth of information available in a genome browser such as UCSC [27]. To get such an effort started, the experimentally validated regulatory elements of the three key transcription factors Oct4, Sox2 and Nanog will be described in this paper. Using the UCSC browser, we can then discuss their evolutionary history. Some observations are linked to Carroll's theoretical work, and they will be listed in Table 1. As we will see, despite incomplete and inaccurate data and a complex phenotype, the computational study of the evolution of gene regulation relevant for stem cells/pluripotency confirms observations from the literature and reveals some interesting insights as well.

**Table 1:** Carroll's 11 principles and the pluripotency genes of the case study.

	Oct4/Pou5f1	Sox2	Nanog
"Mosaic pleiotropy", "Heterotopy"	Role in early embryonic cells, germ cells.	Role in early embryonic cells, germ cells, neural development.	Role in early embryonic cells, somite organization.

"Ancestral genetic complexity", "Deep homology"	All three genes are involved in vertebrate development; cooperation of POU and Sox factors is implicated in bilaterian development (fruit fly and vertebrate).		

"Functional equivalence of distant homologs"	Various 'rescue experiments' in mice, e.g. using Oct4 from chicken [85], frog & axolotl [86].	?	?

"Infrequent toolkit gene duplication"	At most one paralog (pou2/pouv in monotremes & marsupials; Pou5f2 in rodents & primates).	Two close paralogs not expressed in the early embryo (Sox1, Sox3; [87]); three more remote paralogs that may substitute in Oct4 binding (Sox4, Sox11, Sox15).	No known close paralogs.

"Modularity of cis-regulatory elements"	Different roles of the distal and the proximal element.	Different regulatory elements in early embryonic vs. neural tissues.	?

"Vast regulatory networks"	All three genes are part of the large regulatory network underlying pluripotency; see [24,25,88].		

## Methods

### Literature-curated Data and UCSC Conservation and Alignment Tracks

**Figure 1:**
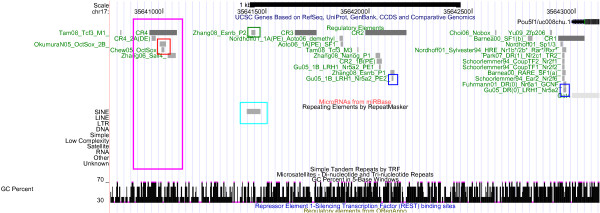
**The Oct4/Pou5f1 Regulatory Region: Regulation and Repeats**. The Oct4/Pou5f1 regulatory region, displayed using the UCSC genome browser [27]. The information on the experimentally validated regulatory elements is displayed below the genome coordinates, using grey blocks and green text in a format consisting of an abbreviated reference (see Table 2), the name of the transcription factor (as it appears in the reference) & its standardized name (if they are not identical), and a specific identifier assigned in the reference (e.g. M1 and M3 distinguishing the two Tfc3 binding sites). Also, the conserved regions CR1 to CR4 and the 2A distal element (DE) and the 1B proximal element (PE) are listed [59]. Below these annotations, repeat information and GC content are shown (moreover, no microRNAs, no ORegAnno [28] annotation or Rest binding [80] is available).

**Figure 2:**
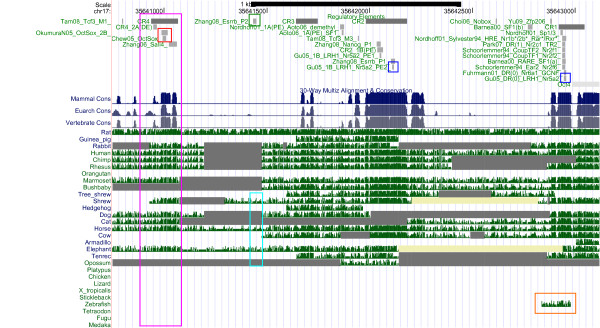
**The Oct4/Pou5f1 Regulatory Region: Conservation**. Below the annotation track (see Figure 1), comparative genomics tracks are displayed. Comparative genomics includes histograms for mammal, Euarchontoglires (rodent, primate, and related species) and vertebrate conservation, and tracks displaying alignment quality as grayscale density. UCSC convention is that yellow regions denote consecutive Ns (lack of sequence) and double lines denote unalignable bases.

**Figure 3:**
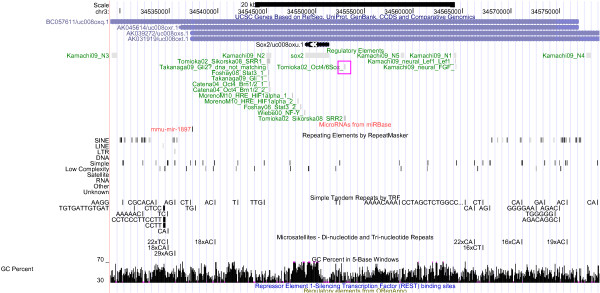
**The Sox2 Regulatory Region: Regulation and Repeats**. The Sox2 regulatory region, displayed using the UCSC genome browser. See Figure 1 for further information. On top, the Sox2 overlapping transcript called BC057611/uc008oxq.1 [81] is visualized, including one microRNA. SRR1 and SRR2 denote conserved regions identified by [82] and [83].

**Figure 4:**
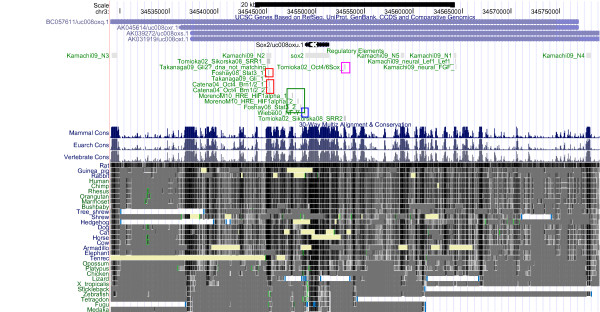
**The Sox2 Regulatory Region: Conservation**. The Sox2 regulatory region, displayed using the UCSC genome browser. See Figure 2 for further information.

**Figure 5:**
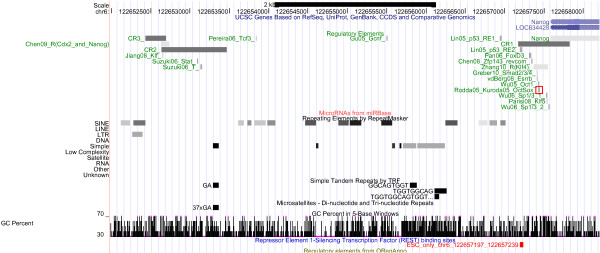
**The Nanog Regulatory Region: Regulation and Repeats**. The Nanog regulatory region, displayed using the UCSC genome browser. See Figure 1 for further information. A REST binding site overlapping a validated p53 binding site is listed. Three conserved regions CR1, CR2 and CR3 [84] are explicitly listed.

**Figure 6:**
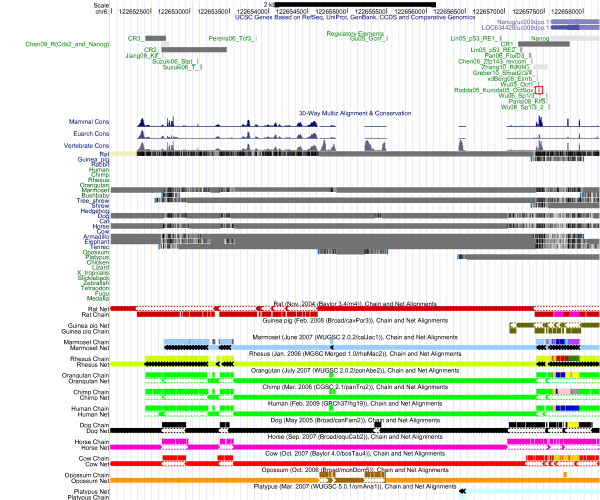
**The Nanog Regulatory Region: Conservation**. The Nanog regulatory region, displayed using the UCSC genome browser. See Figure 1 for further information. Alignment chains and nets are shown because the multiple alignment tracks are very sparse; see the UCSC track documentation for further information about the chains and nets.

To investigate the evolution of gene regulation of the pluripotency factors Oct4 (Pou5f1), Sox2 and Nanog, we first conducted a literature survey of their phylogenetic history and expression patterns. We also assembled a data set of validated TFBSs in the regulatory regions of these genes. Databases of experimentally validated sites in metazoa/vertebrates (such as ORegAnno [28] and Pazar [29]) only cover a small fraction of what is known from the literature. Therefore, an intensive literature search was performed, yielding the TFBS information in Figures 1, 2, 3, 4, 5, 6 (tabulated in Table 2). For each entry marked by '+' in Table 2, we were able to confirm that the nucleotides at the genomic position are indeed the ones reported as the binding site in the corresponding paper. UCSC tracks were generated by formatting the literature data. These tracks can then be viewed together with UCSC multiple alignment and conservation tracks, in three ways:

**Table 2:** Experimentally validated transcription factor binding sites.

Regulatory region of	Transcription Factor Binding Site/Conserved Region	Class of Binding Site/Sub-region	Specific Identifier	Abbreviated Reference	**Ref**.	Chromosomal Position (start/end) ‚+' marks cases where binding site sequence at UCSC and in the reference are identical			
Oct4	Ronin	R()†		Dejosez08	[89]	chr17	35643243	35643348	

Oct4	Nr1b*/2b*; Rar*/Rxr*	HRE		Nordhoff01; Sylvester94	[59,90]	chr17	35642936	35642955	+

Oct4	Nr6a1/GCNF	DR(0)		Fuhrmann01	[91]	chr17	35642943	35642955	+

Oct4	LRH1/Nr5a2	DR(0)		Gu05	[92]	chr17	35642943	35642955	+

Oct4	Nr2c1/TR2	DR(1)		Park07	[93]	chr17	35642936	35642949	+

Oct4	CoupTF1/Nr2f1			Schoorlemmer94	[94]	chr17	35642936	35642949	+

Oct4	CoupTF2/Nr2f2			Schoorlemmer94	[94]	chr17	35642936	35642949	+

Oct4	SF1	RARE	(a)	Barnea00	[95]	chr17	35642936	35642954	+

Oct4	Ear2/Nr2f6			Schoorlemmer94	[94]	chr17	35642936	35642949	+

Oct4	Sp1/3			Nordhoff01	[59]	chr17	35642929	35642939	+

Oct4	Zfp206			Yu09	[96]	chr17	35642877	35642886	+

Oct4	SF1		(b)	Barnea00	[95]	chr17	35642769	35642792	+

Oct4	Nobox			Choi06	[97]	chr17	35642612	35642618	+

Oct4	LRH1/Nr5a2	1B	PE2	Gu05	[92]	chr17	35642109	35642118	+

Oct4	Esrrb		P1	Zhang08	[66]	chr17	35642104	35642123	+

Oct4	Nanog		P1	Zhang08	[66]	chr17	35642033	35642054	+

Oct4	LRH1/Nr5a2	1B	PE1	Gu05	[92]	chr17	35642048	35642057	+

Oct4	Tcf3		M3	Tam08	[98]	chr17	35641942	35641948	+

Oct4	SF1	1A(PE)		Nordhoff01	[99]	chr17	35641863	35641873	+

Oct4	demethylation site	1A(PE)		Aoto06	[59,99]	chr17	35641856	35641875	+

Oct4	Esrrb		P2	Zhang08	[66]	chr17	35641431	35641450	+

Oct4	Sall4			Zhang06	[100]	chr17	35641024	35641063	+

Oct4	OctSox			Chew05	[101]	chr17	35640993	35641008	+

Oct4	OctSox	2B		OkumuraN05	[102]	chr17	35640987	35641016	+

Oct4	Tcf3		M1	Tam08	[98]	chr17	35640793	35640799	+

Oct4	CR2	1B(PE)		Nordhoff01	[59]	chr17	35642037	35642063	

Oct4	CR4	2A(DE)		Nordhoff01	[59]	chr17	35640947	35640965	

Oct4	CR1			Nordhoff01	[59]	chr17	35642918	35643044	

Oct4	CR2			Nordhoff01	[59]	chr17	35641981	35642181	

Oct4	CR3			Nordhoff01	[59]	chr17	35641641	35641746	

Oct4	CR4			Nordhoff01	[59]	chr17	35640937	35641068	

Sox2	NF-Y			Wiebe00	[60]	chr3	34548868	34548886	**+**

Sox2	Lef1, Lef1	neural		Kamachi09	[48]	chr3	34564288	34564307	+

Sox2	FGF	neural		Kamachi09	[48]	chr3	34564324	34564330	+

Sox2	Oct4/6, Sox			Tomioka02	[82]	chr3	34552970	34552985	+

Sox2	Stat3		2	Foshay08	[103]	chr3	34548486	34548498	+

Sox2	Stat3		1	Foshay08	[103]	chr3	34545179	34545199	+

Sox2	Gli		1	Takanaga09	[104]	chr3	34545198	34545206	+

Sox2	Oct4; Brn1/2		1	Catena04	[105]	chr3	34545218	34545229	+

Sox2	Oct4; Brn1/2		2	Catena04	[105]	chr3	34545293	34545302	+

Sox2	HIF2alpha	HRE	1	MorenoM10	[106]	chr3	34547578	34547592	+

Sox2	HIF2alpha	HRE	2	MorenoM10	[106]	chr3	34548182	34548196	+

Sox2	Gli		2 dna_ not_matching^§^	Takanaga09	[104]	chr3	34545074	34545085	

Sox2	N1	neural		Kamachi09	[48]	chr3	34564105	34564403	

Sox2	N2	neural		Kamachi09	[48]	chr3	34544940	34545478	

Sox2	N3	neural		Kamachi09	[48]	chr3	34529134	34529728	

Sox2	N4	neural		Kamachi09	[48]	chr3	34577673	34578140	

Sox2	N5	neural		Kamachi09	[48]	chr3	34558702	34559054	

Nanog	Oct1			Wu05	[107]	chr6	122657427	122657442	+

Nanog	OctSox			Rodda05; Kuroda05	[108,69]	chr6	122657429	122657445	+

Nanog	Smad2/3/4			Greber10	[109]	chr6	122657408	122657412	+

Nanog	Klf4	R()†		Zhang10	[110]	chr6	122657370	122657550	

Nanog	Stat			Suzuki06	[111]	chr6	122653091	122653100	+

Nanog	T			Suzuki06	[111]	chr6	122653135	122653155	+

Nanog	FoxD3			Pan06	[112]	chr6	122657311	122657341	+

Nanog	p53		RE1	Lin05	[113]	chr6	122656935	122656957	+

Nanog	p53		RE2	Lin05	[113]	chr6	122657195	122657221	+

Nanog	Klf			Jiang08	[114]	chr6	122652655	122652663	+

Nanog	Sp1/3		2	Wu06	[115]	chr6	122657555	122657570	+

Nanog	Sp1/3		1	Wu06	[115]	chr6	122657530	122657539	+

Nanog	Tcf3			Pereira06	[116]	chr6	122653837	122653844	+

Nanog	Gcnf/Nr6a1			Gu05	[92]	chr6	122655499	122655511	+

Nanog	Cdx2 and Nanog	R()†		Chen09	[117]	chr6	122652629	122652743	

Nanog	Zfp143	revcom°		Chen08	[118]	chr6	122657340	122657354	+

Nanog	Esrrb			vdBerg08	[119]	chr6	122657409	122657418	+

Nanog	Klf5			Parisi08	[120]	chr6	122657532	122657539	+

Nanog	CR1			Chan09	[84]	chr6	122657170	122657827	

Nanog	CR2			Chan09	[84]	chr6	122652641	122653468	

Nanog	CR3			Chan09	[84]	chr6	122652431	122652696	

† ChIP-defined region

° reverse complement data in paper

§ DNA sequence at UCSC does not match sequence in paper

1) After starting a "Session" from the homepage at http://genome.ucsc.edu/, the "Restore Settings" option in the "Session Management" enables to "Use settings from another user's saved session:". Using "Fuellen" as "user:", "session name:" may be "mm9.Oct4", "mm9.Sox2" and "mm9.Nanog". Alternatively the following links can be used.

• http://genome.ucsc.edu/cgi-bin/hgTracks?hgS_doOtherUser=submit&hgS_otherUserName=Fuellen&hgS_otherUserSessionName=mm9.Oct4

• http://genome.ucsc.edu/cgi-bin/hgTracks?hgS_doOtherUser=submit&hgS_otherUserName=Fuellen&hgS_otherUserSessionName=mm9.Sox2

• http://genome.ucsc.edu/cgi-bin/hgTracks?hgS_doOtherUser=submit&hgS_otherUserName=Fuellen&hgS_otherUserSessionName=mm9.Nanog

2) The "Regulatory Element" tracks are also available as text files in the supplement (Additional Files 1, 2, 3), and can be loaded as custom tracks at the UCSC browser.

3) Using the UCSC genome browser, the gene in question can be located, and the wiki track of its genomic region can be inspected.

All literature-curated TFBS data were also submitted to PAZAR [29,30]. Submission to the ORegAnno [28,31] database has been postponed because the upload facility of ORegAnno was not functional while preparing this manuscript.

### Computational Analysis of the Evolution of Gene Regulation

**Figure 7:**
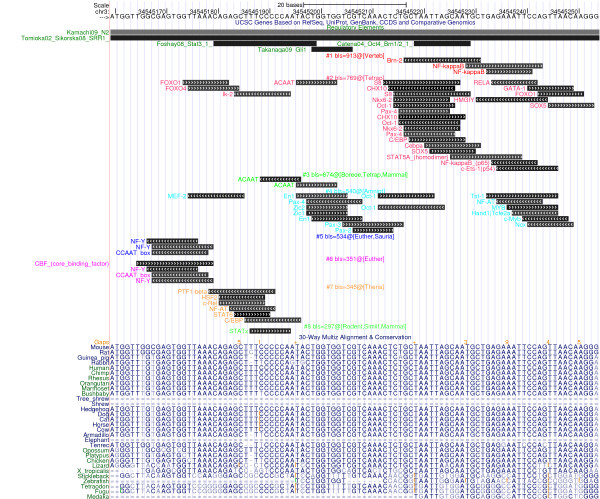
**Part of the Sox2 Regulatory Region, analyzed using ReXSpecies**. Part of the Sox2 regulatory region, displayed using the UCSC genome browser. Extra tracks added by ReXSpecies display the predicted transcription factor binding sites and modules scoring highest.

As described in [12], there are currently a limited number of options available to computationally infer the evolution of gene regulation. In this paper, we focus on the simple approach to study the evolutionary history as described by pre-computed UCSC alignments, and we apply the ReXSpecies software developed in-house. As far as the authors are aware, ReXSpecies is the only tool attempting to directly infer the evolution of gene regulation from the DNA perspective (that is, the gain (and loss) of regulatory elements and modules in phylogenetic history). The first version of ReXSpecies was published in 2008 [32]. Conserved homologous sequences from different species are fetched from UCSC and aligned. In this alignment, transcription factor binding sites (TFBS) are searched using position specific scoring matrices, employing PoSSuM [33,34] and matrix libraries (JASPAR [35] and Transfac [36]). Two TFBSs are considered to be homologs, if they are predicted to be bound by transcription factors known to be homologous, and share essentially the same genomic coordinates. Then, the leaves of a phylogenetic species tree [37-39] are labeled with the TFBS data and the labels of the inner nodes of the tree are estimated using parsimony [40]. Extending the work of 2008, putative modules (groups of TFBSs) are identified based on these inner node labels. These are sets of TFBSs that are predicted to be gained (or lost) at the same inner node of the species tree, and they are then scored using the branch length score as proposed by [41]. Finally, we generate a UCSC annotation track, displaying the modules identified. ReXSpecies was used to generate Figure 7, "Part of the Sox2 regulatory region, analyzed using ReXSpecies."

### Ensembl Gene Trees

Evolution of Oct4, Sox2 and Nanog is studied using gene trees provided by the Ensembl Compara pipeline [42]. The UCSC browser provides a direct link to the same gene at Ensembl, where mouse-over of the gene enables a popup window in which a visualization of the gene can be started. From there, we obtained the Ensembl Compara gene tree using the link called "Gene Tree (image)", on the left.

### Gene Expression Data

We inspected all four UCSC sets of tracks visualizing gene expression data that are available in the UCSC mm9 tracks (GNF Expression Atlas 2[43], GNF Expression Atlas on Mouse Affymetrix U74A Chip, GNF Expression Atlas on Mouse Affymetrix U74B Chip, and GNF Expression Atlas on Mouse Affymetrix U74C Chip [44]). Unfortunately, the first track is not yet documented very well; there is no legend for the relation between color and expression level. The only hint in the track description is: "As is standard with microarray data red indicates overexpression in the tissue, and green indicates underexpression". For the latter three tracks, color is based on a logarithmic scale: "In full mode, the color of each item represents the log base 2 ratio of the signal of that particular experiment to the median signal of all experiments for that probe."

## Results and Discussion

### Oct4, Sox2 and Nanog Evolution

Investigating the regulatory evolution of a set of genes, it is first of interest to know their evolutionary history. The founding father of the POU5 subfamily of POU transcription factors, and ancestor of Oct4/Pou5f1, appears in the lineage of the gnathostomes (jawed vertebrates) [45,46], which includes fish. Two duplicates of the gene (Pou5f1 and pou2/POUV) can be found in tetrapods, but usually one duplicate got lost in today's species; the only known exceptions are monotremes and marsupials [46]. Also, there is a paralog designated POU5F2 in some mammals (rodents and primates), which is involved in sperm development (in case of mouse). The POU5 subfamily is probably most closely related to the POU2 and POU3 subfamilies [47]. The Ensembl gene tree (Additional File 4; Supplementary Figure S1) of Pou5f1 does not consider the paralogs in monotremes and marsupials (the single genes are highlighted by a red box), nor does it consider the POU5 paralogs found in chicken, lizard, frog and axolotl reported in the literature [46]. Instead, the human pseudogene POU5F1P1 and some questionable predicted paralogs in rabbit, guinea pig, kangaroo rat, marmoset, cow, elephant, and armadillo are included in the tree, marked by red duplication nodes. Not considering them, the gene tree suggests that duplications of Pou5f1 are less frequent than thought [46].

Sox2 diverged from its putatively closest paralog Sox1 after the second round of genomic duplication within the vertebrate lineage [48], even though the entire Sox family is probably of metazoan origin [49]. In the Ensembl gene tree (Additional File 5; Supplementary Figure S2), Sox2 evolution is mostly concordant with Carroll's principle of "Infrequent toolkit gene duplication"; the only putative paralogs are Q6WNU1 (in takifugu), Sox14 (in chicken) and some genes around Sox5 (in rat). In the later two cases, we observe long branches (dashed lines, highlighted in red).

Nanog probably originated in the vertebrate lineage [50]; since then it has diverged significantly from its closest paralogs, the NK domain proteins. Apart from the Nanog P1 pseudogene (in human, chimp and gorilla, highlighted in red) and questionable predicted paralogs (all of them highlighted in blue) in some of the monkey genomes (marmoset, orangutan, chimp, gorilla) and in tenrec *(Echinops telfairi)*, guinea pig *(Cavia porcellus)*, and zebra finch *(Taeniopygia guttata)*, the Nanog gene tree at Ensembl (Additional File 6; Supplementary Figure S3) follows species phylogeny and confirms that Nanog duplications are infrequent as well. Sanchez-Sanchez et al [51] suggest that the cooperation of Oct4, Sox2 and Nanog is conserved between mammals and medaka fish; the role of the POU/Sox transcription factor complex in development may even go back to the common ancestor of vertebrates and insects (bilateria) [52,53] (Table 1), a putative case of "Ancestral genetic complexity" and "Deep homology". In case of Oct4, "Functional equivalence of distant homologues" is also documented (Table 1).

### Oct4, Sox2 and Nanog Expression

To summarize the expression of Oct4, Sox2 and Nanog, we refer to recent reviews by Bosnali et al [53] and Johnson et al [54]. Oct4 is restricted to embryonic pluripotent cells of specific stages of development, i.e. the morula, inner cell mass, the primitive ectoderm (epiblast) of the blastocyst, and to cells of the germline. Exemplifying the "modularity of cis-regulatory elements" (Table 1), the Oct/Sox element of the distal enhancer (in the CR4 region, Figure 1, highlighted in red) is deemed responsible for its expression in the morula, inner cell mass and in germ cells, while proximal regulation by the LHR-1 binding sites (in the CR2 and CR1 regions, Figure 1, highlighted in blue) is implicated in its expression in the primitive ectoderm (also known as epiblast), see [54]. Bindings by other factors are scattered across both distal and proximal elements. Nanog is also expressed in embryonic pluripotent cells and germ cells and it plays a role in somite organization [55]. Sox2 expression overlaps with the expression of Oct4 and Nanog, but it also plays a role in adult stem cells of the neural lineage [48], a case of "mosaic pleiotropy", "heterotopy" and "modularity of cis-regulatory elements" (Table 1). Masui et al [56] found that Sox4, Sox11 and Sox15 overlap Sox2 in its expression pattern and are able to replace Sox2 in some of its functionality in embryonic pluripotent cells. In summary, all three genes may be labeled control genes of pluripotency and early development. Accordingly, their regulation shares some, but not all, characteristics of developmental control genes [57]. In particular, they seem to be regulated by a medium number of enhancers (three known clusters of binding sites in case of Oct4, two known clusters in case of Sox2 and Nanog, see Figures 1 to 6) and by microRNAs [58]. All three genes lack a TATA box [59-61] which fits well with the low expression divergence associated with TATA-less genes [62]. Overall, gene expression data displayed at UCSC (see methods) do not reflect what is known from the literature (Additional Files  7,8 and 9; Supplementary Figures S7-S9), since few embryonic data are included at UCSC. Sox2 neural expression (in cerebellum/brain) is most likely true positive.

### Evolution of Pluripotency Core Regulation

Given that Oct4, Sox2 and Nanog can be traced back to the ancestral vertebrate lineage, it can be expected that part of the regulatory elements of Oct4, Sox2 and Nanog are 'pvCNEs', pan-vertebrate conserved noncoding elements [63]. As we can see from Figures 1 to 6, in case of Oct4 and Sox2, a few traces of conserved noncoding elements can indeed be found in fish, based on the UCSC [27] 30-way Multiz alignment & conservation (which includes fish).

### Evolution of Oct4 Regulation (Figures 1 and 2)

The 30-way Multiz alignment at UCSC suggests that the Oct4/Pou5f1 proximal promoter is conserved in jawed vertebrates, since it is found in eutherians and in zebrafish (orange box in Figure 2). Concordantly, Parvin et al [64] describe the zebrafish pou2 proximal promoter, including putative Octamer motifs (which may be bound by pou2) and retinoic-acid responsive elements (which may be bound by nuclear receptors). According to Parvin et al [64], no 'meaningful sequence similarities' between the upstream sequences of pou2 and Oct4 can be identified, though. UCSC data support that the proximal enhancer (CR2 region) is conserved in eutheria and marsupials, and the distal enhancer (CR4 region, highlighted in pink) is conserved at least in eutheria. A recent publication [46] reports the existence of two CR4-like regions in platypus, but only one of them contains a conserved Oct-Sox binding site. No such CR4-like region is displayed at UCSC. Nevertheless, the auto-regulation of Oct4 by itself (and Sox2) is probably a feature shared at least by mammals: Most recently this hypothesis was also put forward by [65]. Inspection of the UCSC RepeatMasker tracks of the regulatory regions of Oct4 indicates that its autoregulation region does not seem to be affected by repeats, cf. Figure 1, pink box. (The specific ERVK repeat retrotransposing Oct/Sox binding sites [1] is included in the RepeatMasker library, but it does not show up here). Interestingly, one Esrrb site (Esrrb_P2, [66], highlighted in green) is found in mammals but not in primates, in line with the observation that Esrrb is not expressed in human embryonic stem cells [67]. Thus, our analysis suggests the loss of a binding site that may be the result of a loss of expression of the transcription factor that binds. Moreover, the Esrrb_P2 site is also the only validated binding site in the Oct4 regulatory region that is part of a repeat identified by RepeatMasker (Figure 1, cyan box). According to UCSC, the repetitive element is a PB1D7 Alu SINE, which originated before the divergence of the primate and the rodent lineages [68]. Inspecting the conservation track, we see some conservation of the Esrrb_P2 site in shrew, horse and elephant (Figure 2, cyan box), so the repeat may indeed be of mammalian origin.

### Evolution of Sox2 Regulation (Figures 3 and 4)

Sox2 is the gene with the most conserved regulatory region (according to UCSC), and it exemplifies best Carroll's principles of "Modularity of cis-regulatory elements", as well as "Mosaic pleiotropy", "Heterotopy", "Ancestral genetic complexity", and "Deep homology" (Table 1). Four upstream conserved subregions are found in mammals, chicken, frog and fish; they can be traced back approx. 500 million years. These conserved regions include the N2 region involved in neural regulation [48] as well as in pluripotency (including validated Stat3 and Oct4/Brn1/2 binding sites, see Figure 4, highlighted in red), and the region around the NF-Y binding site (blue box in Figure 4) of the proximal promoter, just upstream of the transcription start site. The other regions involved in pluripotency, around the downstream auto-regulatory Oct/Sox binding site (pink box) and the proximal Stat3 and HIF1alpha binding sites (green box in Figure 4), are found conserved up to platypus, whereas the other regions involved in neural development (N3, N4, N5) are also found in fish (N1 can be traced back to Xenopus frog). Thus, the hypothesis emerges that neural regulation of Sox2 is as old or older than regulation implicated in pluripotency. There is no evidence that the downstream autoregulatory binding site is affected by repeats, see Figure 3 (pink box). Most of the other validated binding sites are also not part of a repeat identified by RepeatMasker.

An investigation of a subregion of the N2 region, around the experimentally validated Stat3, Gli, and Oct4/Brn1/2 binding sites upstream of Sox2, by ReXSpecies highlights the predicted binding sites and modules displayed in Figure 7. The conserved Stat3 and the Brn1/2 binding sites are among the hits; there is no binding site model for Gli that gives a match. The eight hits are sorted by branch length score (see methods). The module scoring highest is found in track #1, and it is composed of the Brn1/2 binding site and a close-by predicted NFkappaB binding site, both inferred by parsimony to arise at the vertebrate root of the tree. Further predicted modules in tracks #2 and #4 are inferred to have originated in the tetrapod and amniote lineage, respectively. They contain many overlapping predicted binding sites, and possibly at most one of them is valid. Nevertheless, tracks #2 and #4 trigger some interest because they include both pluripotency-related (Oct/Zic) and neural-development-related transcription factor binding sites (Pax/Ncx). These high-scoring tracks may reflect the dual role already noted for the N2 region investigated here. Tracks #3 and #5 display predicted sites/modules with a history of gain and loss inferred by parsimony. The track #3 module was gained in tetrapods and mammals, lost in eutherians and re-gained in boreoeutherians. The track #5 module was gained independently in Eutheria and in Sauria. Tracks #6 and #7 display modules predicted for eutherians and therians, respectively. Finally, the parsimony-based reconstruction of binding site evolution infers that the Stat3 site (track #8, matching the STATx binding site model) was gained in mammals, lost in eutheria, and re-gained in rodents and monkeys (Simiiformes). Whether any of these predicted sites/modules reflect true positive binding (and subsequent regulatory effect) must of course be validated experimentally.

### Evolution of Nanog Regulation (Figures 5 and 6)

At UCSC, the Nanog upstream region does not feature a high-coverage 30-way Multiz alignment (see Figure 6); in case of human, chimp, orangutan, rhesus and cow, individual alignment chains compensate. As noted by Kuroda et al [69] for the OctSox site in the proximal promoter (highlighted in red), conserved regions are shared with elephant (and armadillo & tenrec), so they originated ***before ***the three mammalian superorders (Afrotheria, Xenarthra, Boreoeutheria) split [70,71], about 120 million years ago. The OctSox site is not part of a repeat, but the more distal Nanog upstream region contains a lot of repeats (SINEs, Simple repeats) which partially overlap with the validated binding sites (see Figure 5).

## Conclusions

Genome Browsers such as the UCSC browser are well suited to enable data integration. In our case, combining information already available (on sequence conservation and repeats) with information gained from the literature (on regulatory elements) enabled us to further our understanding of the evolutionary history of some regulatory elements involved in pluripotency. Recently, the UCSC browser started a Wiki track system, and we hope that our effort contributes to a community effort of adding useful information to the system, so that more and more information can be viewed in context, e.g. in the context of conservation and homology information derived from sequence alignment. In particular, it would be useful to have validated regulatory information available for all genes in the genome, for mouse and human alike. To avoid clutter, we suggest that this information is placed into a dedicated "TFBS Wiki" track. (In fact, the Wiki track system should eventually support not just community input to pre-specified Wiki tracks, but it should permit modifications to its structure, such that a hierarchy of tracks can evolve, reflecting the needs of the community.) Moreover, it would be useful to combine such information with network data. For example, validated transcription factor binding sites may directly suggest a link from the transcription factor to its target if both are included in a publicly available network or pathway. Finally, an integration of Wiki projects (UCSC Wiki tracks, WikiGenes [72], WikiPathways [73], and more) may be a worthwhile future goal, enabling community-driven integrative bioinformatics on a large scale, towards a seamless in-silico assembly of knowledge soon after it is obtained on the bench.

## Competing interests

The authors declare that they have no competing interests.

## Authors' contributions

GF wrote parts of the paper and did most of the literature study. SS wrote parts of the paper, generated the figures, managed the data upload to the annotation databases and did the computational studies. Both authors read and approved the final manuscript.

## Reviewers' comments

### Reviewers' report 1

Dr. Gustavo Glusman and Dr. Juan Caballero, Institute for Systems Biology, Seattle, USA(nominated by Dr. Doron Lancet, Department of Molecular Genetics, Weizmann Institute of Science, Rehovot, Israel)

In this manuscript the authors describe a computational analysis of three central pluripotency factors, starting from an extensive literature search for data not available in public databases, and leading to hypotheses about the evolutionary history of the regulation of these genes. The authors present a methodology to integrate external data into the UCSC genome browser. This integration improves the insights that we can infer from different sources, specially using the visualization framework. To validate the method, the authors performed an analysis of the cis-regulatory elements (CREs) present in the promoter regions of the pluripotency-related genes Oct4, Sox2 and Nanog. The integration of conservation data revels patterns of common regulators between multi-species. Also, they propose a molecular history of these regulator in evolutionary time. The authors conclude with a call for community contributions to the novel UCSC Wiki Track system. Due to the nature of the work, this article contains a wide variety of elements. It has many more references than the typical Research Article, almost becoming a mini review. It presents novel untested hypotheses (which might fit the Hypothesis article format) but these are based on more than just a survey of previous results. It reports some specific discoveries made by computational analyses, and thus might fit the Discovery note format, but this would require dropping significant review content. It presents and exemplifies a working methodology that other researchers could emulate, but falls short of such a Tutorial level by relying on pre-computed gene trees, and not describing how to improve on them where they are recognized to be deficient. Finally, its call for community contributions to the UCSC Wiki Track is not accompanied by considerations on the usability of such unstructured content.

#### Authors' Response

We added a more thorough discussion of the pros and cons of the Wiki approach towards the end of the article. In particular, we note that a specific "Wiki TFBS" track should be established by UCSC (see also below). We give a more detailed description of the points we want to get across, and how the structure of the article follows from this, by adding another paragraph of the introduction as follows:

In summary, we wish to:

*1) Exemplify how the UCSC browser can be used to investigate the evolution ofgene regulation*.

*2) Exemplify how the Wiki track at UCSC could be used to support suchinvestigations by a large-scale community effort*.

*3) Report the results we obtained from our study of the evolution of generegulation of three specific genes*.

*4) Put our results into a wider, general context by referring to Carroll's theoretical work*.

While the need to address such distinct aspects of the work is understandable, the intermediate format currently used caused a loss of focus on the most important aspect(s) of the work. Too much importance seems to be given to the methodology used, which is not an original method: the power of data integration in bioinformatics and systems biology is well known. The UCSC genome browser and many other genomic browsers allow the integration of personal and external data sources, and have APIs to facilitate this. On the other hand, a deeper analysis and discussion of the evolutionary history of the cis-regulators for 3 key genes in pluripotency could be more important for the reader and the scientific community. The manuscript could be shortened, or perhaps restructured by moving the less central content to the (currently very short) Methods section. Reformatting the article to regain focus would also help clarify the figure set. At the moment, the first figure referred to in the Results section is Figure 9, with the first six figures introduced in the Methods. Figure 8, its associated legend text and reference 76 are never referenced in the manuscript - in fact, it's not clear why the BED format, never mentioned in the text, would need to be illustrated by a figure in this paper.

#### Authors' Response

*We removed the figure explaining the BED format and renumbered the figures*.

In the Methods section, it is claimed that "Databases of experimentally validated sites in metazoa/vertebrates (such as ORegAnno and Pazar) only cover a small fraction of what is known from the literature", which necessitated the extensive literature search that was performed. The results of the literature search were summarized in the Supplementary Table 1. It would be interesting to show, in that table, which of the sites identified in the literature search were already annotated in ORegAnno and Pazar. Were this indeed a small fraction? Were there sites in the databases that were not recovered by the literature search that was performed? Do the authors have confidence that the literature search was extensive enough? In that Supplementary Table, sites marked with '+' could be identified with confidence because the nucleotide sequences mentioned in the articles is identical to that in the reference genome. How were the other sites treated?

The authors mention that data submission to ORegAnno was "postponed because the upload facility of ORegAnno was not functional while preparing this manuscript". If this is a temporary technical difficulty with that database, is it of use to the reader to know of the delay?

#### Authors' Response

*At the time of writing, Pazar featured two of the binding sites we compiled, and ORegAnno did not feature any. The literature references associated with the Pazar sites were followed up, validated and included. We believe that the literature search converged based on the observation that for Oct4/Pou5f1, two recent reviews (Niwa, 2007 *[74] and Kang et al, 2009 [75]) *list a subset of the sites we found, but no additional ones. Sites that could not be validated (nucleotide sequences mentioned in the articles are not identical to that in the reference genome) were included and marked clearly (as regions, denoted "R()" in the table). The problems with OregAnno are unfortunately persistent. More specifically, before our annotation work, the UCSC ORegAnno track did not contain any entries in the regions that we investigated. Also, we did not find any entries via the ORegAnno web site*.

*Pazar lists some entries in the corresponding regions in three projects ("TFe", "Pleiades genes", and "Pluripotency", the latter is our contribution). Most of the "TFe" and the "Pleiades genes" entries refer to regions larger than 150 base pairs. In these cases, our entries are an improvement, because they contain the exact position of TFBSs. For Sox2, there are entries for the regions N3 and N4. For Pou5f1, one match for NR2F2 is listed with a PubMed reference *[76], *which overlaps with our entry from *[77]. *The six other annotations for Sox2 are longer than 150 bp. For Nanog, one of our annotations, the Sox2 part of the heterodimer TFBS that we have called "Oct4 Sox2" reported by Rodda *[78]* and by Kuroda *[79]*already existed, split in two entries (one for each author) in the "Pleiades genes" project. Two predictions of more than 150 bp in length can also be found*.

Presumably because of this technical difficulty, the authors added the regulatory sites identified via literature search to the UCSC Wiki Track, and suggest this as a role model for other researchers to emulate. Is this really a good idea, though? The UCSC browser already includes a large number of tracks for specific types of genomic information, and most users will naturally search for TFBS data in TFBS tracks (like ORegAnno). Would it not be counterproductive for researchers to default to adding their analysis results to the Wiki Track, instead of attempting to add them to the more relevant tracks? What would the Wiki Track look like if thousands of researchers added to it a pot pourri of different data types, many of them redundant with existing tracks? At which point would the Wiki Track lose its usability, and by being a catch-all, would the other tracks become less reliable in their completeness if researchers opt to dump data into the Wiki Track instead?

#### Authors' Response

*As stated towards the end of the article, we believe that a wiki-based information resource can keep up best with the large amount of data being generated. But we agree that the Wiki track must be subdivided, so that our information must go into a "Wiki TFBS" track. Upon publication of the article, we will approach the UCSC Genome browser people with respect to this issue*.

We added the following text to the last paragraph of the article:

To avoid clutter, we suggest that this information is placed into a dedicated "TFBS Wiki" track. (In fact, the Wiki track system should eventually support not just community input to pre-specified Wiki tracks, but it should permit modifications to its structure, such that a hierarchy of tracks can evolve, reflecting the needs of the community.)

Additional comments by section

Abstract, 1st paragraph

"Experimentally validated data on gene regulation are hard to obtain."

This claim is unclear, as there are many ways to obtain gene regulation information (i.e. microarrays, ChIP-seq).

#### Authors' Response

*ChIP-seq only considers binding, no regulatory effect. ChIP-seq combined with microarray data is a high-throughput approach that delivers data of lower quality, as compared to the direct small-scale experiments in the papers that we tracked down*.

3rd paragraph

"Based on the expected return on investment for the community, [...]"

The conclusions didn't mention the insights obtained from the promoter analysis and evolutionary conservation in the promoter regions of the pluripotency-related genes.

#### Authors' Response

We now start the conclusions as follows:

*We were able to elucidate some aspects of the evolution of gene regulation for three genes associated with pluripotency*.

Background, 2nd paragraph

"If transcription factors bind to (some of) these elements, the amount of transcription may be altered."

The dynamic of the interactions between TF-CREs is vaguely described, besides the regulation of the regulators is not presented (i.e. TF translocation, phosphorilation cascades, miRNA regulation of the TF).

#### Authors' Response

*We write "may be altered" for good reason. Giving more details would indeed turn this part of the text into a mini-review*.

"Closest to the transcription start site are the core and the proximal promoter, followed by distal elements."

This need a reference and some coordinates or lengths to describe the typical order and size of the sub-regions in a promoter.

#### Authors' Response

*We now write "Next to the transcription start site are the core and the proximal promoter (up to 250 base pairs), followed by the distal elements (the latter are more than 250 base pairs away from the transcription start site)*[4].*"*

"Many regulatory elements evolve due to mutations, insertions and deletions of nucleotides (by selection, or by random drift), or due to transposable elements."

Also transposable elements can import new CREs into a promoter region, and other variation events can occur affecting the regulatory region (duplication, inversion, translocation).

#### Authors' Response

We now write "Many regulatory elements evolve due to mutations, insertions and deletions of nucleotides, by selection, duplication, inversion, translocation or by random drift, or due to transposable elements."

3rd paragraph

"Genes that have to be regulated synchronously are expected to share some or all of their cis-regulatory elements." and "Concentrating on the subnetwork of early development, the challenge becomes smaller, even though due to the pleiotropy, subnetworks in general are not independent."

Missing references.

#### Authors' Response

*While most of these two claims is self-evident, we now cite Carroll here*.

4th paragraph

"Assuming that conservation goes with functional importance, "phylogenetic profiling"‚ and related methods [13-22]..."

The number of references could be reduced; 3-4 tool examples would be enough.

Methods, 1st paragraph

Supplemental Table 1 could be improved by including the consensus sequence for each motif and the observed sequence in the promoter.

"The 'Regulatory Element' tracks are also available as text files in the supplement, and can be loaded as custom tracks at the UCSC browser."

Supplement number/title is missing.

#### Authors' Response

*We added Supplement number/title*.

2nd paragraph

"Two TFBSs are considered to be homologs, if they are predicted to be bound by transcription factors known to be homologous, and share essentially the same genomic coordinates."

This need not be enough evidence of homology, as there are many ambiguous and unspecific matrix motifs that produce high rate of false positives. Many phylogenetic profiling methods use a filtering step calculating a p-value or entropy of the motif detected using a control dataset of sequences.

#### Authors' Response

*Correct, but the prediction method is supposed to take care of this issue; we consider the E-values provided by the prediction method*.

Results and discussion, 1st paragraph

"Oct4, Sox2 and Nanog evolution"

Evolutionary time could help to understand the origin and duplication history in all the cases presented.

#### Authors' Response

*It's future work to take a close look at evolutionary time. However, we expect that there is no "molecular clock", so insights may be limited*.

How do the authors define a "questionable predicted paralog"?

#### Authors' Response

*A questionable predicted paralog is a sequence that is likely due to miss-prediction or miss-assembly. Evidence for this is that the species is not among the standard species for which gene predictions have been validated extensively, and are based on a high-coverage genome assembly. Further, the sequence is usually included in the EnsEmbl gene tree together with another sequence from the same species carrying the canonical name such as "Sox2"*.

2nd paragraph

"In particular, they seem to be regulated by a medium number of enhancers (three known clusters of binding sites in case of Oct4, two known clusters in case of Sox2 and Nanog, see Figures 1 to 6) and by microRNAs [58]."

A regulatory diagram could help to visualize the regulation of the 3 genes.

4th paragraph

"Thus, our analysis suggests the loss of a binding site that may be the result of a loss of expression of the transcription factor that binds."

Or a change in the sequence specificity of the TF, or a TF substitution.

#### Authors' Response

*Correct, it's correlation (of loss of binding site and loss of expression of the transcription factor that binds), **not **causality, so the interpretation is indeed just a plausible suggestion*.

5th paragraph

"Evolution of Sox2"

A comparison of the conservation of CREs in the other family members could expand the view of the regulation of Sox2.

## Conclusions

Again, the conclusion is focused exclusively in the methodology used, not in the insight gained in the promoter analysis of Oct4, Sox2 and Nanog.

References

The number of references could be significantly reduced for a research article.

Figures

Figure 8 is unnecessary.

### Authors' Response

*See above*.

Supplemental files

The figures showing expression values need a title and description besides a scale for the expression levels/colours relationship.

### Authors' Response

*We added these*.

We declare that we have no competing interests.

Gustavo Glusman & Juan Caballero

Institute for Systems Biology

### Reviewer's report 2

Dr. Niels Grabe, TIGA Center (BIOQUANT) and Medical Systems Biology Group, Institute of Medical Biometry and Informatics, University Hospital Heidelberg, Germany(nominated by Dr. Mikhail Gelfand, Department of Bioinformatics, Institute of Information Transfer Problems Russian Academy of Science, Moscow, Russian Federation)

Previously, it has been shown that the transcription factors Oct4, Sox2 and Nanog are of key importance in cellular pluripotency. The authors demonstrate how DNA binding sites for these transcription factors, which are extracted from literature, can be further analyzed with the UCSC genome browser system. For the question of the evolution of gene regulatory elements alignments of the binding sites were performed and introduced into the system. The authors generally propose the scientific community to use the UCSC in combination with wiki approach to collect experimental TF binding sites.

### Comments

1.) Title: I am not sure in how far the sub-title "the case for wiki tracks at the UCSC" is easily understandable to a broader readership.

#### Authors' Response

*We modified the title, now writing "the case for wiki tracks at genome browsers." For the intended readership, we believe this is more understandable*.

2.) The Abstract should be improved for a broader readership: The authors should make clearer what wiki and custom tracks are. The fact that multiple alignments have been performed should be included in the abstract. Also the authors should be more specific in how far what Carroll's thesis are and in how far they have been confirmed.

#### Authors' Response

We amended the "Results" section of the abstract as follows:

*We demonstrate the power of integrative bioinformatics by including curated transcription factor binding site information into the UCSC genome browser, using wiki and custom tracks, which enable easy publication of annotation data. Data integration allows to investigate the evolution of gene regulation of the pluripotency-associated genes Oct4, Sox2 and Nanog. For the first time, experimentally validated transcription factor binding sites in the regulatory regions of all three genes were assembled together based on manual curation ofdata from 39 publications. Using the UCSC genome browser, these data were then visualized in the context of multi-species conservation based on genomic alignment. We confirm previous hypotheses regarding the evolutionary age of specific regulatory patterns, establishing their 'deep homology'. We also confirm some other principles of Carroll's 'Genetic theory of Morphological Evolution', such as "mosaic pleiotropy", exemplified by the dual role of Sox2 reflected in its regulatory region*.

3.) Methods section: Paragraph "Literature curated data": I would suggest to transfer details of the UCSC handling in 1), 2) and 3) into the supplements of the manuscript and restrict the descriptions to the general overall idea.

#### Authors' Response

*Since there is no page limit, we believe that the text should be as self-contained as possible*.

4.) Methods section: Paragraph "Computational analyses": The general strategy used here should be made clearer. For example it is not clear whether ReXSpecies has actually been used or not.

#### Authors' Response

Indeed, this paragraph was not clear at all. At the beginning of the paragraph, we now write:

*"As described in *[12], *there are currently a limited number of options available to computationally infer the evolution of gene regulation. In this paper, we focus on the simple approach to study the evolutionary history as described by pre-computed UCSC alignments, and we apply the ReXSpecies software developed in-house. As far as the authors are aware, ReXSpecies is the only tool attempting to directly infer the evolution of gene regulation from the DNA perspective (that is, the gain (and loss) of regulatory elements and modules in phylogenetic history). The first version of ReXSpecies was published [...]"*

At the end of the paragraph, we added the clarification that:

*"ReXSpecies was used to generate *Figure 7, *"Part of the Sox2 regulatory region, analyzed using ReXSpecies.""*

5.) Results section: As the manually collected binding sites are central importance for the manuscript, the authors should think about including the supplementary tables in the main document.

#### Authors' Response

*We included the supplementary table in the main document*.

### Reviewers' report 3

Review by Dr. Franz-Josef Müller, Center for Regenerative Medicine, The Scripps Research Institute, La Jolla, CA, USA and University Hospital for Psychiatry and Psychotherapy (part of ZIP gGmbH), University of Kiel, Germany(nominated by Dr. Trey Ideker, University of California San Diego, La Jolla CA, United States).

Fuellen and Struckmann combine proposing a crowd-sourcing approach to annotate transcription factor binding sites (TFBS) with a more specific analysis of TFBS evolution of pluripotency associated transcription factors.

Both topics are interesting, yet the combination proves to be problematic since the resulting review/hypothesis/data paper hybrid seems to be less succinct and stringent than I would wish for in a scientific manuscript. I do think, that rigorous focus on fewer key point and a significant shortening of the manuscript and reduction of the figure count will benefit the manuscript.

#### Authors' Response

*We believe that the combination is well-justified: Just proposing the Wiki approach without highlighting its benefits would not be convincing. However, based on the other reviews, we added a clear list of aims at the end of the "Background" section and we believe that this new text addresses the concern of "rigorous focus". Also, we reduced the number of figures by moving the three figures regarding the UCSC expression data and the gene trees into the Supplement*.

There are also issues in regard to the main hypothesis: while the conclusion, that curation efforts such as in a wiki-track in the scientific community would be highly desirable, there is currently no realistically viable system how such an effort could be supported in our current high impact and grant driven system.

#### Authors' Response

*We agree on this "political" issue. But we believe that something should be done now, and that our paper may get the ball rolling after all. In the medium term, we believe that it must be a condition for acceptance of a scientific paper that main results are made available to the community in a form that is community-editable and, if possible, computer-readable (and we would like to stress that the Wiki idea includes track-keeping of all modifications, so that a common knowledgebase is created that includes a "history" enabling credit assignment). In the long term, we believe that community/wiki resources and scientific publications will converge into a single multi-faceted interconnected resource*.

Would, for example, I put a postdoc on such a curation effort project?

Most likely not, because how could she/he become an independent researcher with publications that are 'just' metadata curation efforts, which most likely will not be accepted in any conventional original research journals. Although it would be desirable in ideal world that such efforts would be adequately honored, it is not likely that this will happen anytime soon. The alternative model is that companies take up the task and professional curation of literature findings is a pay for service. The most prominent example is BioBase, which offers the Transfac database for researchers at a reduced fee (~$3000), which is much less than a postdoc/year.

I really don't want to get into the copy left/copy right discussion, I do believe that information should be free and accessible, especially if its generation was funded by taxpayers money supporting non-profit research, but still we have to acknowledge the imperfections in our scientific systems and how human beings act in it. Thus I would like to ask the authors to discuss and also compare commercial databases (Transfac is actually pretty good for the analysis of pluripotent stem cells) as an alternative and where the authors see their concept in regard to such existing concepts.

#### Authors' Response

*Since access to commercial databases is limited, we do not wish to perform such a comparison. Also, such a comparison would be problematic because there are companies offering similar services. We think that there is room for large-community efforts as well as for commercial data-curation efforts; in particular we expect commercial data-curation efforts to be more focussed on specific topics (e.g. disease-related data)*.

One may argue, that such databases will not contain TFBS information for, say gorillas, but are such information actually relevant beyond a focused study such as the one by Fuellen and Struckmann?

There are currently several, yet unpublished efforts under way to reprogram endangered species (sometimes with only 7 individuals left on this planet). The main problem, these researchers are facing are actually not unknown TFBS, but to be able to use the genomic sequences of the reprogramming factors so these can be cloned for the reprogramming vectors since these species are usually not sequenced.

Looking at this from this angle, shouldn't we instead make the case for more high quality sequencing of other species and improvements in our (functionally relevant) TFBS-prediction algorithms for an instant online prediction of such sites, if specific question arise?

#### Authors' Response

We hope that resources can be allocated to both efforts!

## Acknowledgements and Funding

Funding by the DFG SPP 1356, 'Pluripotency and Cellular Reprogramming' (FU583/2-1) and by the BMBF (01GN0901, Generation of pluri- and multipotent stem cells) is gratefully acknowledged. Nitesh Singh helped with some of the figures. Clemens Harder verified the annotations that we uploaded to UCSC and Pazar. Sherry Freiesleben revised the annotations that were uploaded to Pazar.

## Supplementary Material

Additional file 1 **The annotations for Oct4 (also listed in Table **2**) as file in BED format, which can be uploaded to genome browsers**. Click here for file

Additional file 2 **The annotations for Sox2 (also listed in Table **2**) as file in BED format, which can be uploaded to genome browsers**. Click here for file

Additional file 3 **The annotations Nanog (also listed in Table **2**) as file in BED format, which can be uploaded to genome browsers.** Click here for file

Additional file 4 **Supplementary Figure S1 - Gene Tree of Pou5f1.** Ensembl gene tree of Pou5f1. Click here for file

Additional file 5 **Supplementary Figure S2 - Gene Tree of Sox2. **Ensembl gene tree of Sox2. Click here for file

Additional file 6 **Supplementary Figure S3 - Gene Tree of Nanog.** Ensembl gene tree of Nanog Click here for file

Additional file 7 **Supplementary Figure S7 - Gene Expression tracks for Pou5f1.** Gene expression tracks at the UCSC genome browser for the murine Pou5f1 gene. Green color indicates underexpression, red overexpression. Click here for file

Additional file 8 **Supplementary Figure S8 - Gene Expression tracks for Sox2.** Gene expression tracks at the UCSC genome browser for the murine Sox2 gene. Green color indicates underexpression, red overexpression. Click here for file

Additional file 9 **Supplementary Figure S9 - Gene Expression tracks for Nanog.** Gene expression tracks at the UCSC genome browser for the murine Nanog gene. Green color indicates underexpression, red overexpression. Click here for file
