# Supplementary figures and images for: Evolution of gene regulation of pluripotency - the case for wiki tracks at genome browsers
Source: Biol Direct. 2010 Dec 29;5:67. doi: 10.1186/1745-6150-5-67 (PMC3024949; doi:10.1186/1745-6150-5-67)

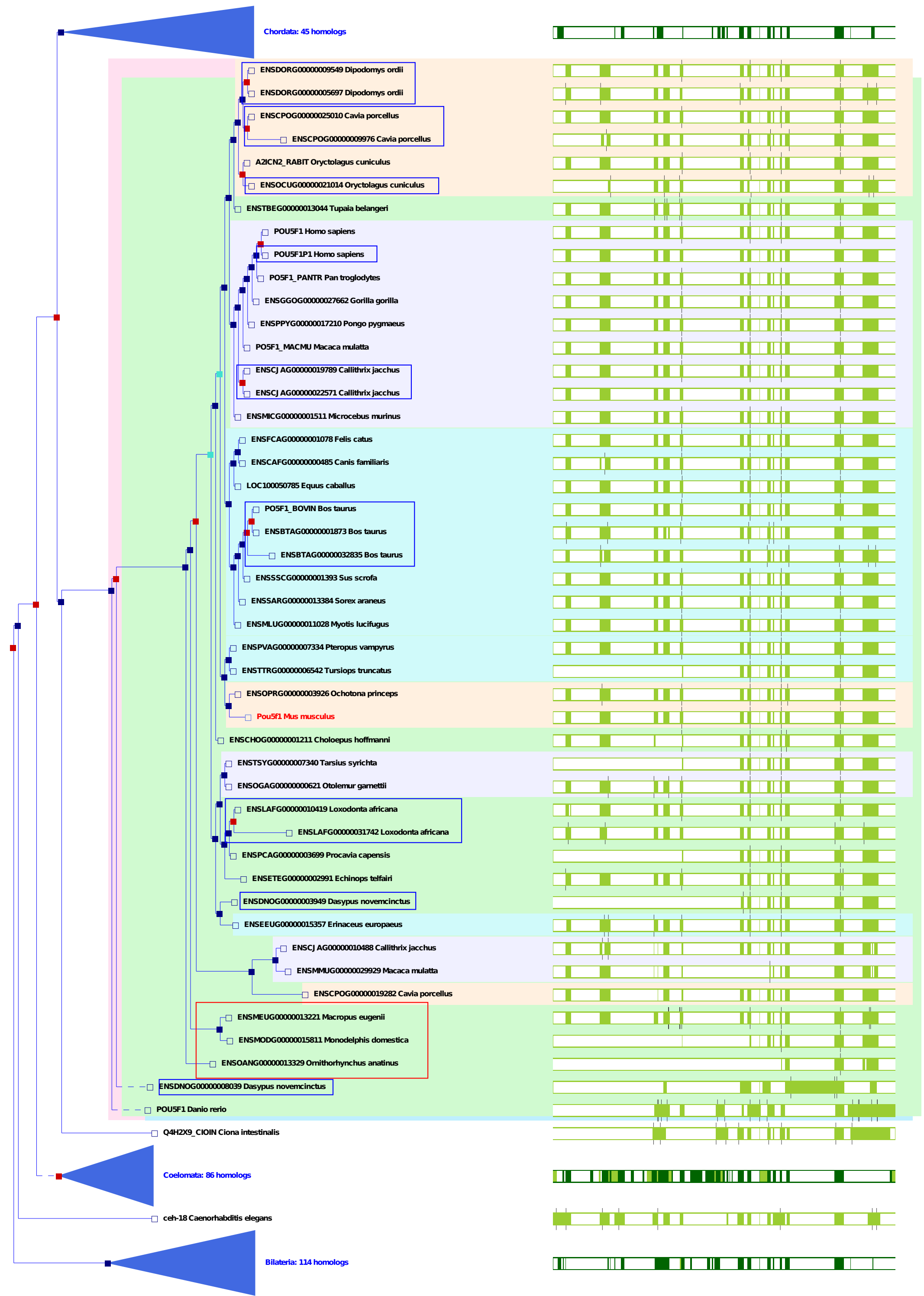

Supplement: Additional file 4 — Supplementary Figure S1 - Gene Tree of Pou5f1. Ensembl gene tree of Pou5f1. [file 1745-6150-5-67-S4.PDF]

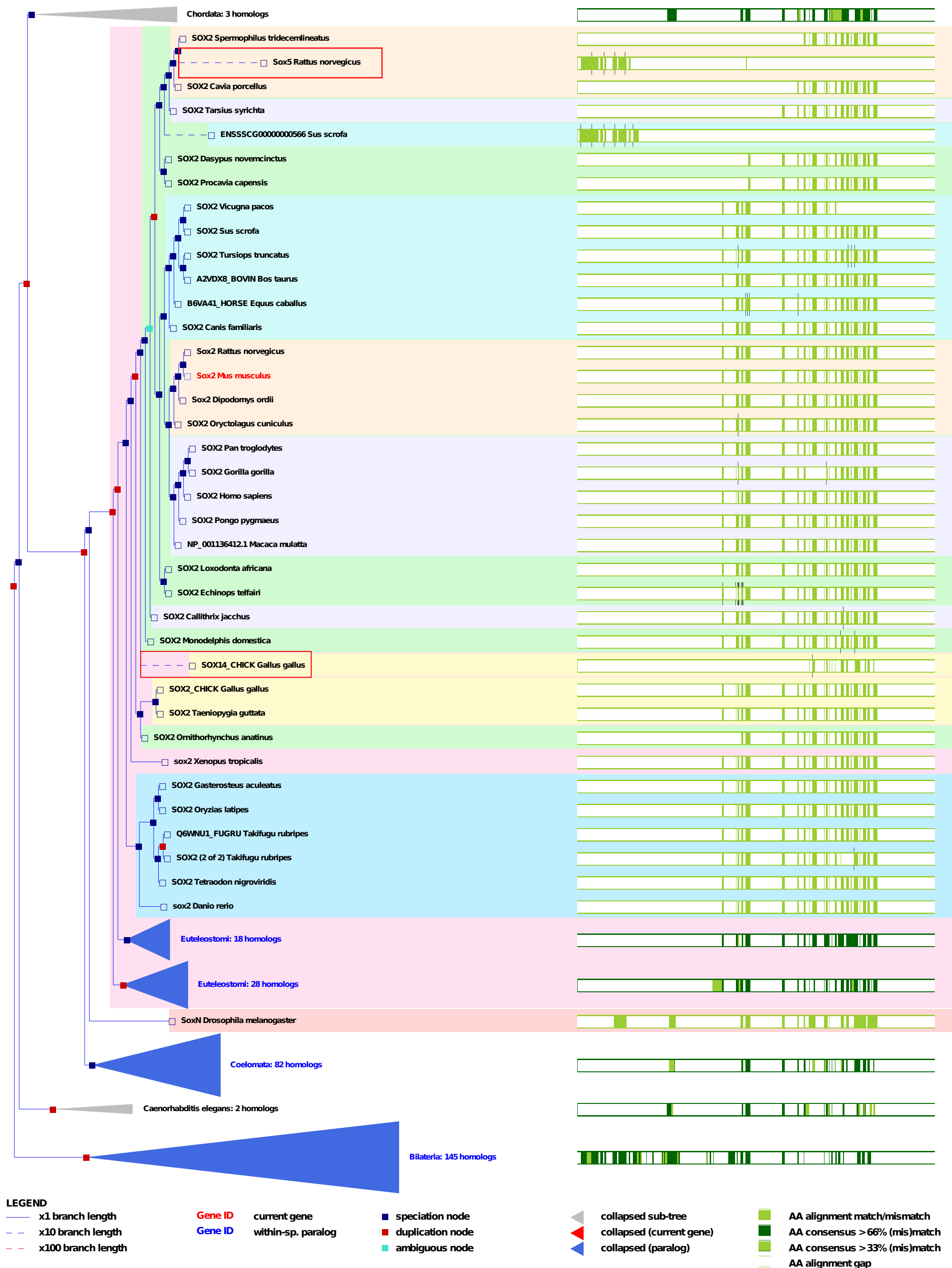

Supplement: Additional file 5 — Supplementary Figure S2 - Gene Tree of Sox2. Ensembl gene tree of Sox2. [file 1745-6150-5-67-S5.PDF]

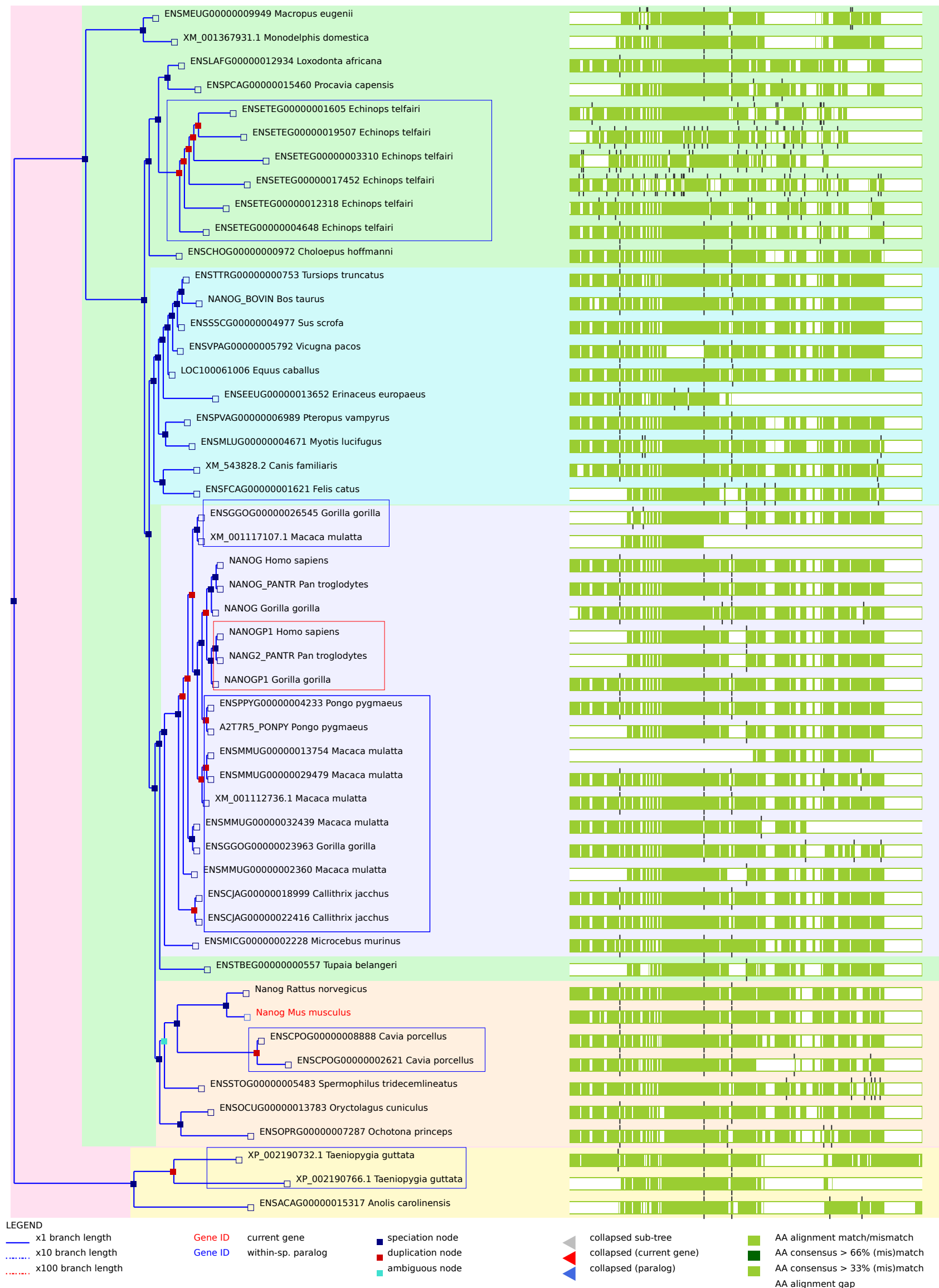

Supplement: Additional file 6 — Supplementary Figure S3 - Gene Tree of Nanog. Ensembl gene tree of Nanog [file 1745-6150-5-67-S6.PDF]

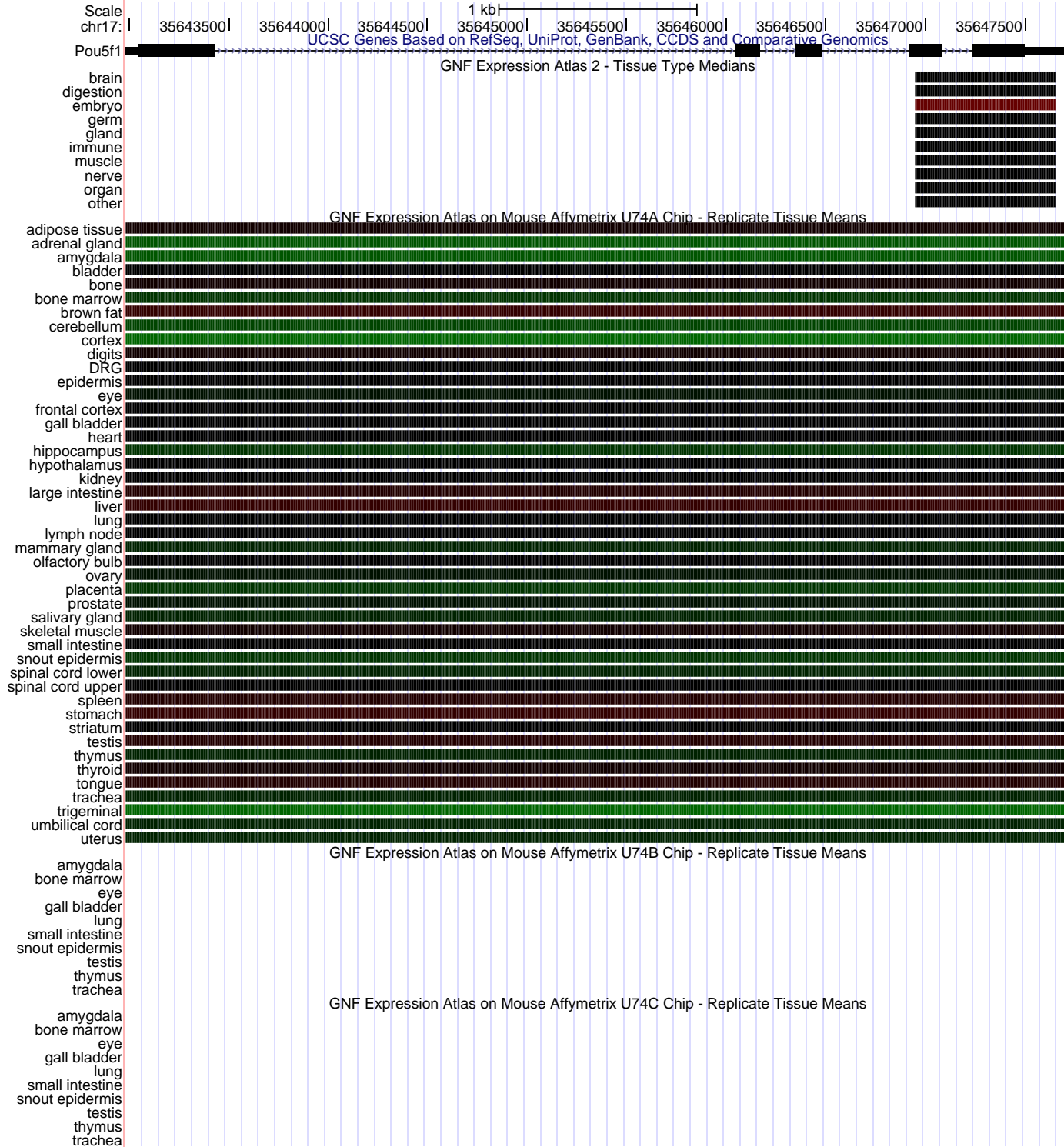

Supplement: Additional file 7 — Supplementary Figure S7 - Gene Expression tracks for Pou5f1. Gene expression tracks at the UCSC genome browser for the murine Pou5f1 gene. Green color indicates underexpression, red overexpression. [file 1745-6150-5-67-S7.PDF]

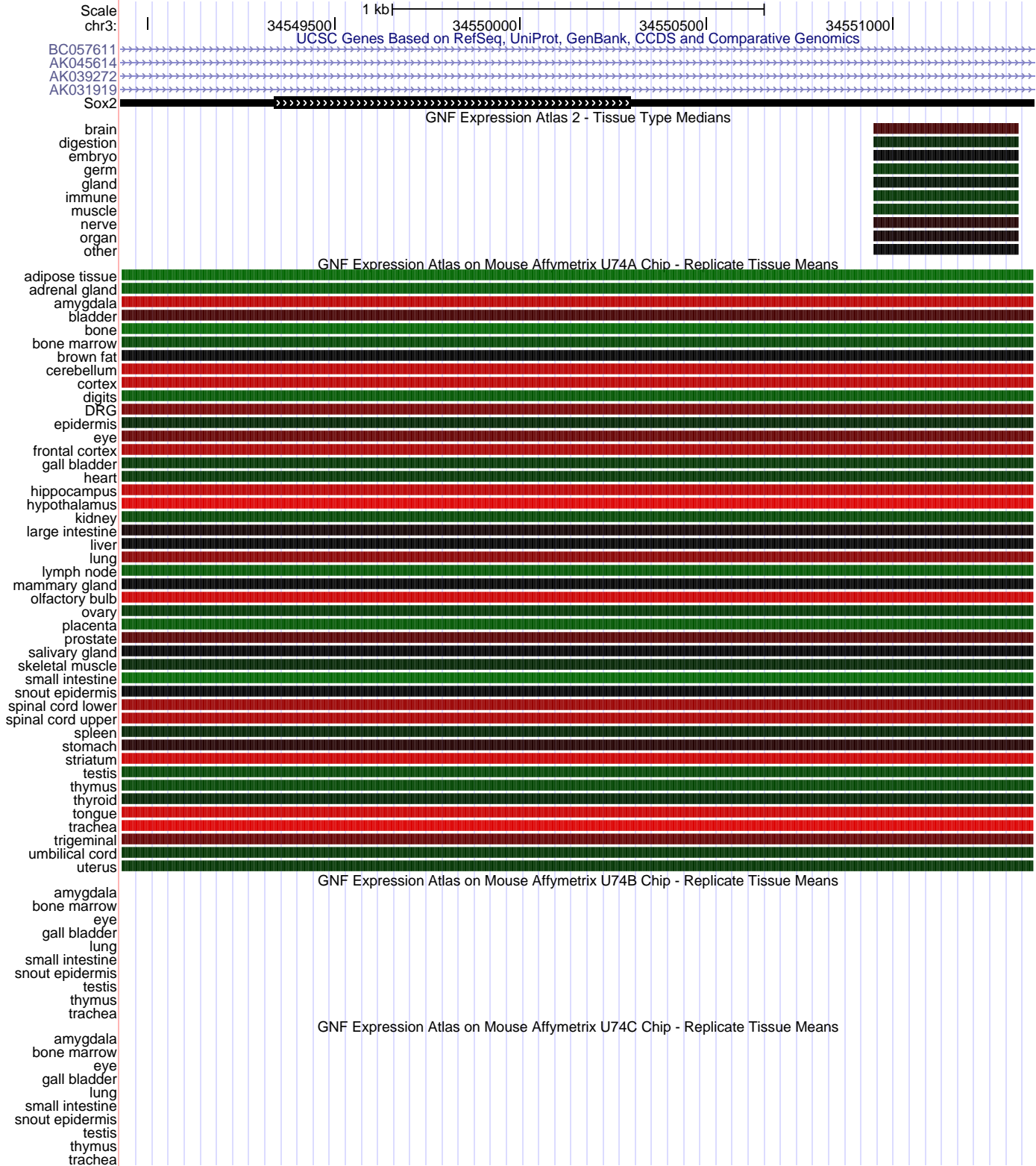

Supplement: Additional file 8 — Supplementary Figure S8 - Gene Expression tracks for Sox2. Gene expression tracks at the UCSC genome browser for the murine Sox2 gene. Green color indicates underexpression, red overexpression. [file 1745-6150-5-67-S8.PDF]

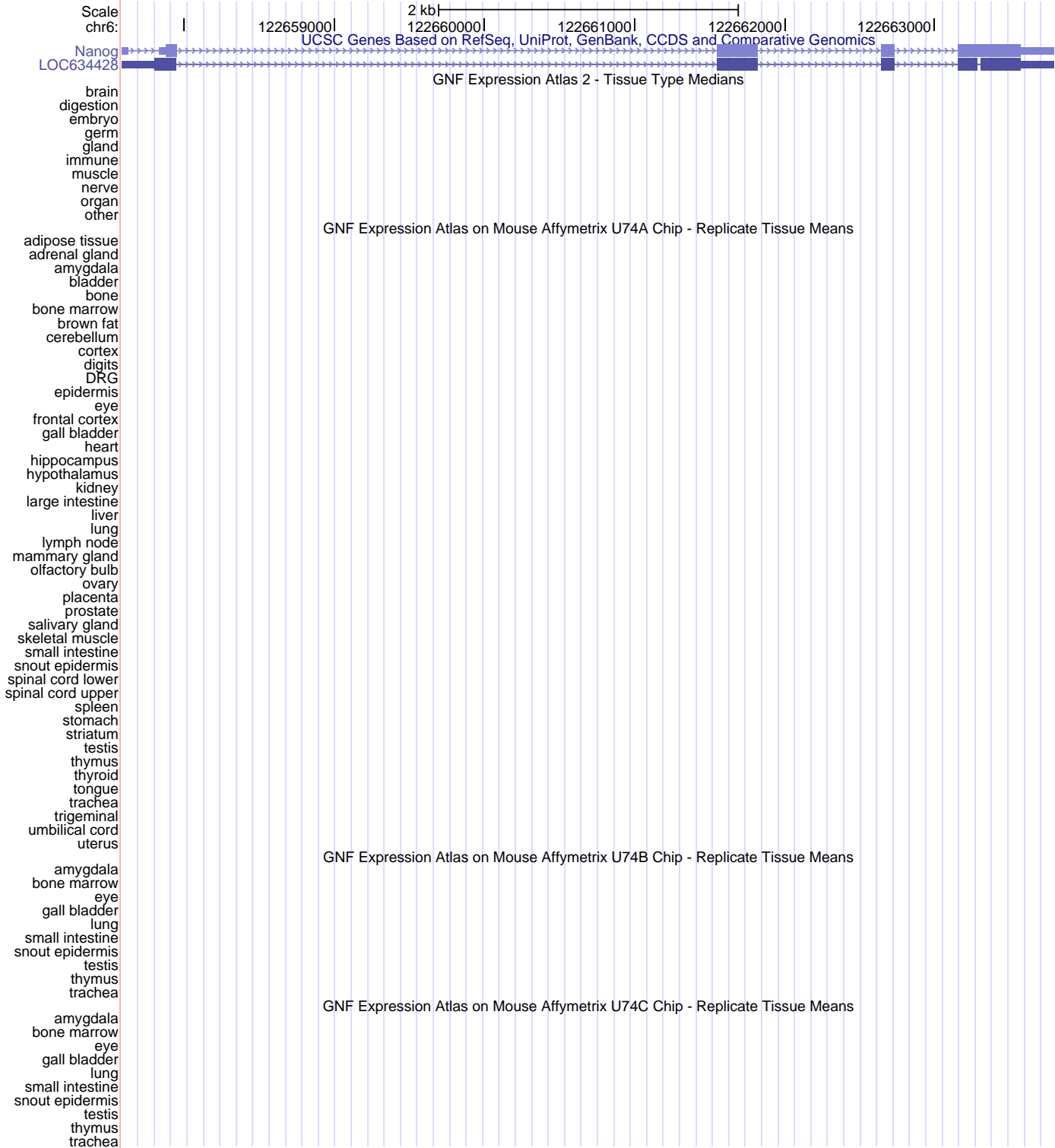

Supplement: Additional file 9 — Supplementary Figure S9 - Gene Expression tracks for Nanog. Gene expression tracks at the UCSC genome browser for the murine Nanog gene. Green color indicates underexpression, red overexpression. [file 1745-6150-5-67-S9.PDF]
